# Prescription reporting with immediate medication utilization mapping (PRIMUM): development of an alert to improve narcotic prescribing

**DOI:** 10.1186/s12911-016-0352-x

**Published:** 2016-08-22

**Authors:** Rachel B. Seymour, Daniel Leas, Meghan K. Wally, Joseph R. Hsu, the PRIMUM Group

**Affiliations:** 1 Department of Orthopaedic Surgery, Carolinas Health Care System, 1000 Blythe Boulevard, Charlotte, 28203 NC USA; 2 Carolinas Trauma Network Research Center of Excellence, Carolinas Health Care System, 1320 Scott Avenue, Charlotte, NC 28204 USA

**Keywords:** Opioids, Electronic medical record, Prescription drug abuse, Clinical decision support

## Abstract

**Background:**

Prescription narcotic overdoses and abuse have reached alarming numbers. To address this epidemic, integrated clinical decision support within the electronic medical record (EMR) to impact prescribing behavior was developed and tested.

**Methods:**

A multidisciplinary Expert Panel identified risk factors for misuse, abuse, or diversion of opioids or benzodiazepines through literature reviews and consensus building for inclusion in a rule within the EMR. We ran the rule “silently” to test the rule and collect baseline data.

**Results:**

Five criteria were programmed to trigger the alert; based on data collected during a “silent” phase, thresholds for triggers were modified. The alert would have fired in 21.75 % of prescribing encounters (1.30 % of all encounters; *n* = 9998), suggesting the alert will have a low prescriber burden yet capture a significant number of at-risk patients.

**Conclusions:**

While the use of the EMR to provide clinical decision support is not new, utilizing it to develop and test an intervention is novel. We successfully built an alert system to address narcotic prescribing by providing critical, objective information at the point of care. The silent phase data were useful to appropriately tune the alert and obtain support for widespread implementation. Future healthcare initiatives can utilize similar methodology to collect data prospectively via the electronic medical record to inform the development, delivery, and evaluation of interventions.

## Background

Increases in opioid prescriptions for acute and chronic pain have led to rises in abuse and overdose of prescription narcotics [1]. Unintentional poisonings due to opioid abuse and misuse is an ever growing and significant cause of death and injury. While opioids are the most common class of scheduled medications involved in deaths related to pharmaceutical overdose (75.2 %), benzodiazepines are involved in nearly a third of these overdoses (29.4 %). Additionally, the combination of opioids and benzodiazepines is especially dangerous, with opioids implicated in 77.2 % of deaths involving benzodiazepines, making these two classes of drugs the target of many interventions [2]. Nearly 15,000 people in the United States die from prescription painkiller overdoses every year [3]. In the past 12 years, the rates of unintentional poisoning deaths have surpassed motor vehicle deaths in many states; specifically, unintentional poisoning deaths in North Carolina have risen 308 % [4, 5].

All levels of government in the United States recognize the crisis of prescription narcotics. The Executive Office of the President recently released a National Plan for Prescription Drug Abuse, detailing goals relative to this problem, including encouraging and even rewarding prescribers who check Prescription Drug Monitoring Programs (PDMP) before writing prescriptions for opioids. The document went further with a call to find ways to utilize the electronic medical record (EMR) to help identify and curb prescription drug abuse [6]. In fact, the 2016 budget includes $133 million in new funding to address opioid misuse and abuse, leading the U.S. Health and Human Services Secretary to announce an initiative focusing on three priority areas—prescriber guidelines, naloxone, and medication-assisted treatment—to address the issue [7]. The Centers for Disease Control and Prevention also recognizes the importance of improving opioid prescribing practices as a strategy to reduce the number of people who misuse, abuse, or overdose from these medications [3].

Opioids and benzodiazepines are considered controlled substances according to the U.S. Drug Enforcement Administration, based on the potential for abuse or dependency [8]. Prescribers in the outpatient setting (primary care, internal medicine, and dentists) generate the majority of prescriptions for these controlled substances [9]. In fact, several sources suggest that a small number of these prescribers generate the vast majority of controlled substance prescriptions [10–12]. Therefore, interventions targeting prescribing behavior holds considerable potential for addressing this epidemic. “First do no harm”, or *Primum non nocere*, is a guiding principle in medicine. *PRIMUM* provides prescribers with critical information to put this principle into practice.

Frequently, prescribers in different care settings are blind to patient interaction with prescribers in other facilities or the critical information that is buried within the overwhelming amount of health information in each patient’s electronic record [13, 14]. Multiple attempts to utilize the EMR and institute computerized physician order entry (CPOE) and computer-based clinical decision support have proven successful. Current alert systems programmed into the EMR are designed to make the prescriber aware of critical information. Health maintenance reminders, allergy alerts, prescription drug interaction alerts, and systems to alert physicians to contraindications due to chronic condition or medications are all imbedded in healthcare systems and physician practices [15–19]. Some of these alerts have resulted in behavior change through increased vaccination rates [20], increased use of prophylaxis among patients at risk for deep-vein thrombosis and pulmonary embolism [21], and reduction of prescriptions for contraindicated agents [17], among others. However, the number of alerts have grown to the point where physicians may see 80 alerts per 100 medications ordered, causing these alerts to be ignored in up to 90 % of cases [22], so it is important to spend a significant amount of time developing and testing an alert to maximize its’ clinical utility. Research on alert systems indicates that systems focusing on critical information that is clear, concise, and timely is most likely to be well-received and have a positive impact [23–25]. Alert triggers must be optimized to focus on the specific population of interest because alerts that interrupt work flow too often introduce “alert fatigue” and will be ignored [24, 26].

We propose using a technological solution – clinical decision support logic built into the EMR—to help address the problem of unintentional poisonings. Clinical decision support has been developed to increase adherence to clinical practice guidelines for opioid therapy for chronic non-cancer pain in the primary care setting, including dosing recommendations and contraindications [27, 28]. However, EMR-integrated alert systems have not previously been used to identify patients with evidence-based risk factors for misuse, abuse, or diversion of prescription narcotics, including both opioids and benzodiazepines. Additionally, we believe this is the first attempt to provide decision support in the acute setting.

A multidisciplinary team within our large healthcare system sought to address the prescription narcotic epidemic. The “Prescription Reporting and Immediate Medication Utilization Mapping” intervention, or PRIMUM, is the result of these efforts. We saw an opportunity to utilize the EMR to map prescription utilization and to provide real-time information to prescribers at the point of care, analyze the data on a continuous basis, and make improvements to the system based on feedback from physicians and other prescribers in our numerous care locations, encompassing outpatient clinics, emergency departments, urgent care centers, and hospitals.

The purpose of our investigation was:

1. To create a rule within the EMR to identify at risk patients utilizing searchable, objective indicators of risk for misuse, abuse, and diversion of prescription controlled substances based on peer-reviewed literature and consensus opinion.
2. To use data generated by the rule in an iterative improvement process to tune the timing of the alert and thresholds for the triggers to produce an acceptable number of relevant alerts at the point of care.

## Methods

A multidisciplinary team developed and implemented an alert system that *provides integrated clinical decision support into the electronic medical record* to impact prescribing behavior and, thereby, reduce prescriptions for opioids and benzodiazepines for at-risk patients to ultimately impact patient outcomes. The development and testing of the rule occurred between December 2014 and May 2015, within our large, integrated healthcare system with a common EMR. A list of terms and definitions related to this alert system is provided in Table 1.

**Table 1:** Definitions

Rule	The rule is executed every time a prescription for an opioid or benzodiazepine is initiated. The rule is programmed to search the medical record for the triggers.
Trigger	Triggers are the criteria the rule searches for in the medical record. These triggers are indicators of risk for opioid or benzodiazepine misuse, abuse, and/or diversion.
Alert	The alert is the pop-up box that appears when the rule identifies the patient to meet one or more of the trigger criteria. The alert interrupts workflow and requires the prescriber to either override the alert and continue with the prescription or cancel the prescription before proceeding.
Patient Encounters	A patient encounter is any medical visit or interaction a patient has with a system provider. This includes inpatient stays, outpatient visits, lab visits, and ED/Urgent Care visits.
Prescribing Encounters	Any patient encounter in which a prescriber initiates a prescription for one or more opioids or benzodiazepines is defined as a prescribing encounter.

### Context

Our healthcare system presents a unique opportunity to test this innovative intervention, with over 40 hospitals, 6 freestanding emergency departments, 28 urgent care locations, and ~800 physician practices with over 15,000 physicians. A common EMR, Cerner [29], is used by a subset of these facilities, and participated in the implementation of PRIMUM. 15 hospitals, 6 freestanding emergency departments, 27 urgent care locations, and 379 physician practices with over 2500 prescribers were included in the PRIMUM implementation and evaluation. The PRIMUM rule was written into the Cerner EMR using a standard rule template included as part of the EMR program. All of the sites utilizing Cerner were included in all phases of the implementation and testing.

#### Aim 1

To create a rule within the EMR to identify at risk patients utilizing searchable, objective indicators of risk for misuse, abuse, and diversion of prescription controlled substances based on peer-reviewed literature and consensus opinion.

### Identification of the risk factors

An Expert Panel of study co-investigators and other key stakeholders, including clinical and research faculty from multiple surgical and medical specialties (Emergency Medicine, Behavioral Health, Orthopaedic Surgery, Internal Medicine, Pharmacy), senior healthcare system physician and administration leadership, Information Services, and experts in the field of unintentional poisoning was convened. Considerable effort was placed on ensuring engagement from physicians, researchers, and all levels of healthcare system administration, including presentations to system-level committees, individual meetings, and grand rounds lectures for physicians across the system. The Expert Panel was charged with selecting criteria that would indicate risk of misuse, abuse, or diversion of prescription narcotics for potential inclusion in a rule built within the EMR. Review of current medical literature was utilized to identify evidence for or against inclusion of these perceived risk factors.

Further extensive literature review conducted by the Expert Panel generated a more expansive list of risk factors associated with misuse, abuse, or diversion of prescription drugs, including demographic characteristics, medical conditions, prescription history and details, and a variety of identified high risk behaviors. A complete listing of all risk factors identified in the peer-reviewed literature is presented in Table 2. Following the systematic review of the literature to outline all potential risk factor, the team worked collaboratively to determine the risk factors to be built into the rule.

**Table 2:** Risk Factors Associated with Misuse, Abuse, or Diversion of Prescription Drugs

Demographic characteristics	*Medical* c*onditions*	*Prescription details*	*High* r*isk* b*ehaviors*
Race (Caucasian [37, 49–52], Non-Hispanic [34, 37], American Indians/Alaskan Natives [34]) High school education or less [49, 53, 54] Age (Younger to middle aged) [34, 37, 53, 55, 56] Not married [37, 50, 53, 54] Financial Problems [37] Unemployed [37] Male [34, 35, 53, 55, 57] Income extremes [34, 53, 55, 57] Rural residence [34, 37] LGBT [57] Public insurance [55] Low social class [58] Family history of substance abuse [59] Preadolescent sexual abuse [59]	Past Suicide Attempt [49] Lifetime Heroin use [49] Pain [39, 49, 50, 54, 60–62] Tobacco use [37, 54] Alcohol use [37–39, 57, 59] Current illicit drug use (including marijuana, cocaine, heroin, methamphetamine, hallucinogens) [37, 39, 57] Physical disability [37] Mental health problems [34, 39, 50, 59, 61, 63] Substance abuse disorder [39, 50, 53, 59] Hepatitis A, B, C [39] Past hospitalization [50] Opioid dependence [50] Liver disease [50] Congestive heart failure [50] Cerebrovascular disease [50] Chronic pulmonary disease [50] Diabetes [50] Hypertension [50] Cancer [50] Cardiovascular disease [50] Obesity [50] Medical comorbidities [55, 63] Past care at psychiatric hospital [58] ADHD [64]	Multiple prescribers [30, 33, 34, 52, 53, 56, 59, 65] Multiple pharmacies [30, 31, 33, 34, 56] High community prescribing rates [34] Treatment with high daily dose opioids and short-acting opioids [39, 50] Multiple prescriptions [31, 35, 50, 52, 56] Overlapping prescriptions [31] High maximum prescribed daily morphine equivalent dose [34, 35, 50, 66, 67] Preexisting opioid use [62] Co-prescribing of opioids and benzodiazepines [34]	Multiple prescribers [30, 33, 34, 52, 53, 56, 59, 65] Multiple pharmacies [30, 33, 34, 56] Multiple ED visits [50, 55, 59] Request for refill [59, 60] Lost or stolen medication [59, 60] Request for parenteral medication [60] Reported allergies to non-narcotic medications [52] Requesting medication by name [52] Weekend visit [52] Use of alias [68] Abnormal urine/blood screen [59] Resist therapy changes/alternative therapy [59] Canceled clinic visits [59]

### Selecting the triggers

To develop an alert based on these risk factors in the EMR, the criteria must be consistently and accurately documented in the EMR in a specific location that can be searched in an automated fashion. In addition, investigators agreed to focus on objective risk characteristics rather than on subjective or anecdotal assessments of patient risk or behavior in order to limit the potential for the rule introducing bias into the medical encounter. The risk factors were mapped against objective data available and searchable in the EMR to determine which risk factors were initially eligible for inclusion. Demographic characteristics and many comorbidities were excluded as potential triggers as they may indicate risk but have low specificity and, while associated, are not on the causal pathway. Given that many of the documented drug-seeking behaviors are more subjective and are not immediately searchable in the EMR, they were not included as triggers. The remaining indicators of risk included prescription details, administration of controlled substances, and information about substance use.

Expert panel discussions of these remaining risk factors yielded 5 categories of objective information available in the EMR that speaks to risk for misuse, abuse, or diversion and that were agreed upon by all members of the panel: current open prescriptions [30–32]; visits to emergency departments or urgent care facilities with onsite treatment with narcotics; 30 day prescription narcotic history [33–35]; history of overdose [36]; and history of positive drug or alcohol screens within the EMR [36–39]. The expert panel came to a consensus on the initial threshold for each trigger based on the literature as well as clinical experience in a variety of specialties with diverse patient populations.

#### Aim 2

To use data generated by the rule in an iterative improvement process to tune the timing of the alert and thresholds for the triggers to produce an acceptable number of relevant alerts at the point of care.

### “Silent” data collection

We used the EMR rule to prospectively collect surveillance data on narcotic prescriptions (defined as opioids or benzodiazepines) within our health system in a series of defined silent periods. This rule would eventually power the alert in the EMR; however, we were able to run it “silently” (no alert displayed to prescribers) in order to collect data needed to create an effective alert. During the silent phase, the rule captured information about each patient encounter in which an opioid or benzodiazepine was prescribed, including the selected triggers, patient identifiers, location, encounter type, prescriber information, and the medication prescribed.

The first data collection period, *Silent Surveillance 1*, lasted 1 month and focused on testing the programming of the rule and report generation. We also reviewed preliminary data on risk characteristics and the rate at which the alert would have been triggered. *Silent Surveillance 2* was conducted for 1 month to ensure the risk criteria were appropriately tuned and to describe the narcotics prescribing patterns across the system.

### Tuning the trigger thresholds

These triggers were included in a rule developed to retrieve these data from the EMR in real-time and present them to the prescriber at the point of care. In order to assure appropriate tuning of the triggers, we used *Silent Surveillance 1* data to revise the triggers to optimize sensitivity and provide meaningful and actionable information to prescribers while minimizing prescriber burden and alert noise [40]. We did not set an a priori alert rate for the triggers. Rather, the panel reviewed the patient populations being captured and missed by the current thresholds and came to a consensus to revise thresholds accordingly. These revised trigger thresholds were monitored during *Silent Surveillance 2*.

### Tuning the timing

The timing of the alert presentation to the prescriber is critical. The information needs to be presented early enough in the encounter to affect prescribing behavior for the at-risk patients. However, if the alert appears too early in the encounter, it could bias the entire patient visit. Some data suggest that the patient-clinician relationship can affect patient outcome [41], so unnecessary bias of this relationship could cause harm. If the alert appears later in the encounter, after the full prescription has been written, it would improve data capture for the purpose of research, but may contribute to work flow disruption within a busy practice or even ignoring the alert since it is after the “point of decision”.

In order to determine the appropriate timing of the alert, we conducted literature reviews, expert panel discussions, and individual interviews with practicing clinicians from across the system to obtain feedback. The individual interviews solicited input from clinicians of varying experience (resident and attending), various specialties (emergency medicine, primary care, surgical subspecialties, etc.) and clinician type (physician and nurse practitioner).

## Results

### Development of the alert

The following alert triggers were initially programmed into the rule through the literature search and the expert panel discussions:

- ◦ Current prescription with >30 % remaining expected/early refill (e.g. A prescription written for 10 days duration was prescribed at least 3 days ago).
- ◦ 3+ visits to ED or Urgent Care with onsite treatment with opioids (not including visits leading to admission) within previous 30 days.
- ◦ 3+ prescriptions for opioids or benzodiazepines within previous 30 days.
- ◦ Previous presentation for overdose within the EMR.
- ◦ Positive screen for blood alcohol, cocaine, or marijuana within the EMR.

**Fig. 1:**
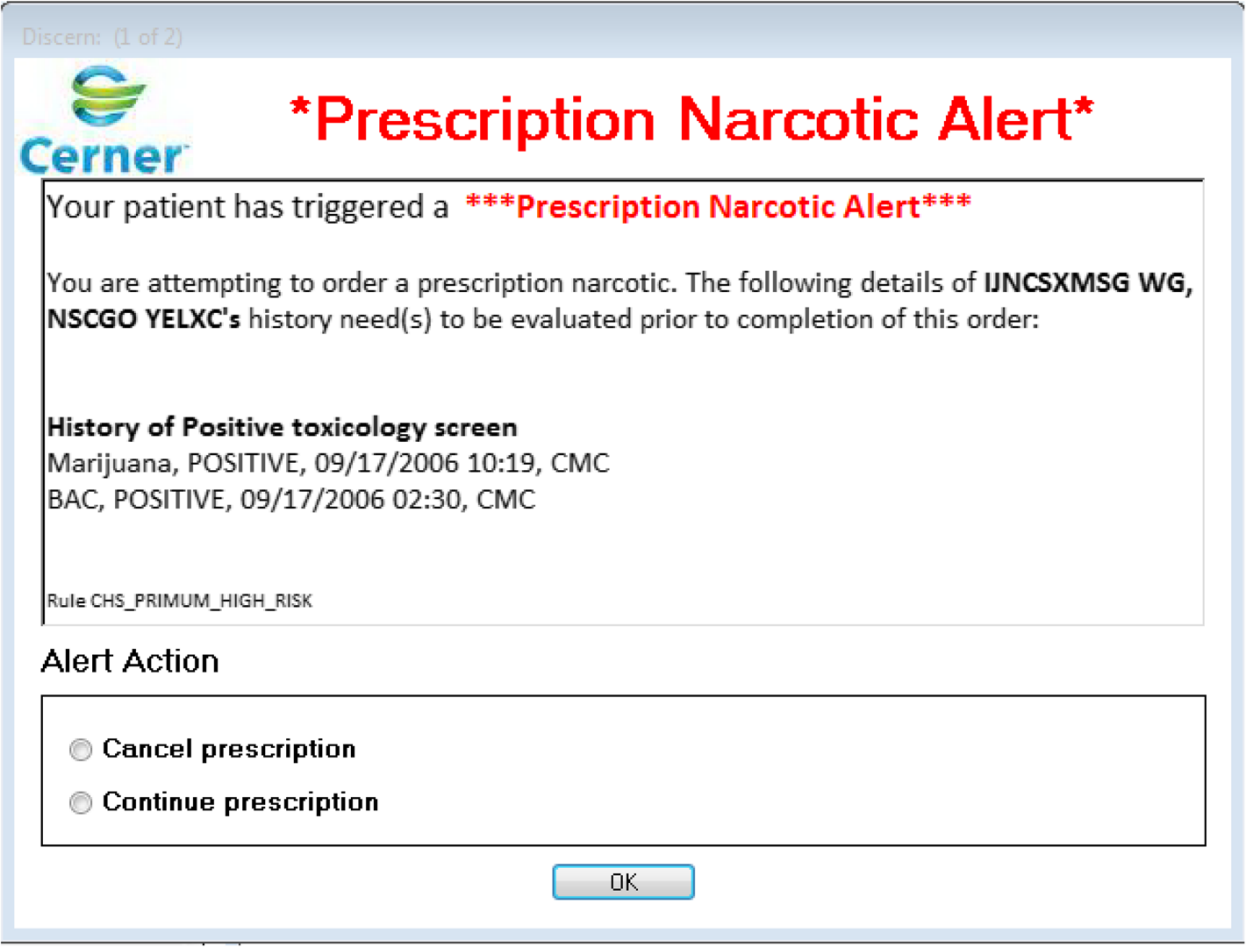
Prescription Narcotic Alert in the EMR

If one or more of the alert triggers are met, once live, an alert fires in the EMR and presents the information to the prescriber (Fig. 1). The alert is concise, clear, and shows details regarding only the triggers that are applicable to that patient [40]. Navigating the alert requires minimal time; the prescriber choses to continue with the prescription as planned or to cancel the prescription.

### Tuning the timing and trigger thresholds

Peer-reviewed literature, consensus panel and individual clinician interviews arrived at placing the alert at the point when the prescriber chooses an opioid or benzodiazepine. This timing was chosen to provide the information as soon as possible and decrease interruption to workflow that would occur if the prescriber received the alert after completing the prescription entirely (i.e. after completing dosage and pill count information or signing the prescription). However, the alert does not appear as soon as the patient record is opened, to avoid labeling patients in encounters where narcotics or benzodiazepines are not involved. The feedback from the expert panel and the clinician interviews was consistent with timing from human factors research on the electronic medical record and avoidance of a negative bias on the encounter [42–45].

After reviewing *Silent Surveillance 1* data, the thresholds of two of the triggers were modified (indicated in bold). The initial “current prescription with >30 % remaining/early refill” trigger was present in approximately 17 % of prescriptions. Upon further review of the patients meeting this criteria, many of the early refills were found to be in primary care offices. Many patients obtain prescriptions for refills when at a regularly scheduled appointment with their physician and, therefore, the initial threshold of 30 % was believed to be too low to appropriately capture the at risk patient. Conversely, the initial “3+ visits to ED or Urgent Care with onsite treatment” trigger was present in only 1.58 % of prescriptions. We determined this rate was low, and we had the potential to identify a greater number of at risk patients without burdening prescribers with high alert rates. The Final Triggers programmed into the rule were:

- ◦ Current prescription with >50 % remaining expected/early refill.
- ◦ 2+ visits to ED or Urgent Care with onsite treatment with opioids (not including visits leading to admission) within previous 30 days.
- ◦ 3+ prescriptions for opioids or benzodiazepines within previous 30 days.
- ◦ Previous presentation for overdose within the EMR.
- ◦ Positive screen for blood alcohol, cocaine, or marijuana within the EMR.

After revision of the trigger thresholds, *Silent Surveillance 2* was conducted, and the rates of alerts and triggers were reviewed (Table 3). By changing the threshold to current prescription with >50 % remaining, the rate of this trigger decreased from 17 % in *Silent Surveillance 1* to 13.54 % in *Silent Surveillance 2*. Decreasing the onsite treatment with narcotics to 2+ visits in the previous 30 days yielded a rate of 1.96 % instead of 1.58 %. Although this change did not increase the rate drastically, the modified alert trigger allowed us to identify over 200 additional patients at risk. These new trigger thresholds were considered appropriately tuned to both identify at risk patients and balance concerns about alert fatigue among prescribers. There were a total of 81,841 prescriptions written during this month in 61,747 prescribing encounters.

**Table 3:** Characteristics of Controlled Substance Prescribing Encounters, Silent Surveillance 2 (*n* = 61,747 prescribing encounters)

Characteristic	No. (% Prescribing encounters)
Age of patient	
< 18	1552 (2.51 %)
18-64	45,571 (73.80 %)
65	14,624 (23.68 %)
Facility type	
ED/Urgent Care	18,267 (29.58 %)
Inpatient Discharge	4656 (7.54 %)
Outpatient including phone calls	38,310 (62.04 %)
Other	514 (0.83 %)
Class of drug	
Opiate	45,165 (73.15 %)
Benzodiazepine	14,268 (23.11 %)
Both	2314 (3.75 %)
Number of criteria met (of any combination)	
0	48,164 (78.00 %)
1	10,517 (17.03 %)
2	2654 (4.30 %)
3	369 (0.60 %)
4	43 (0.07 %)
5	0 (0.00 %)
Criteria met	
Prescription with >50 % remaining	8358 (13.54 %)
2+ visits with onsite administration	1208 (1.96 %)
3+ prescriptions in past 30 days	2873 (4.65 %)
Positive tox screen	4165 (6.75 %)
BAC	1444 (2.34 %)
Cocaine	1248 (2.02 %)
Marijuana	2440 (3.95 %)
Previous presentation for overdose	500 (0.81 %)

**Fig. 2:**
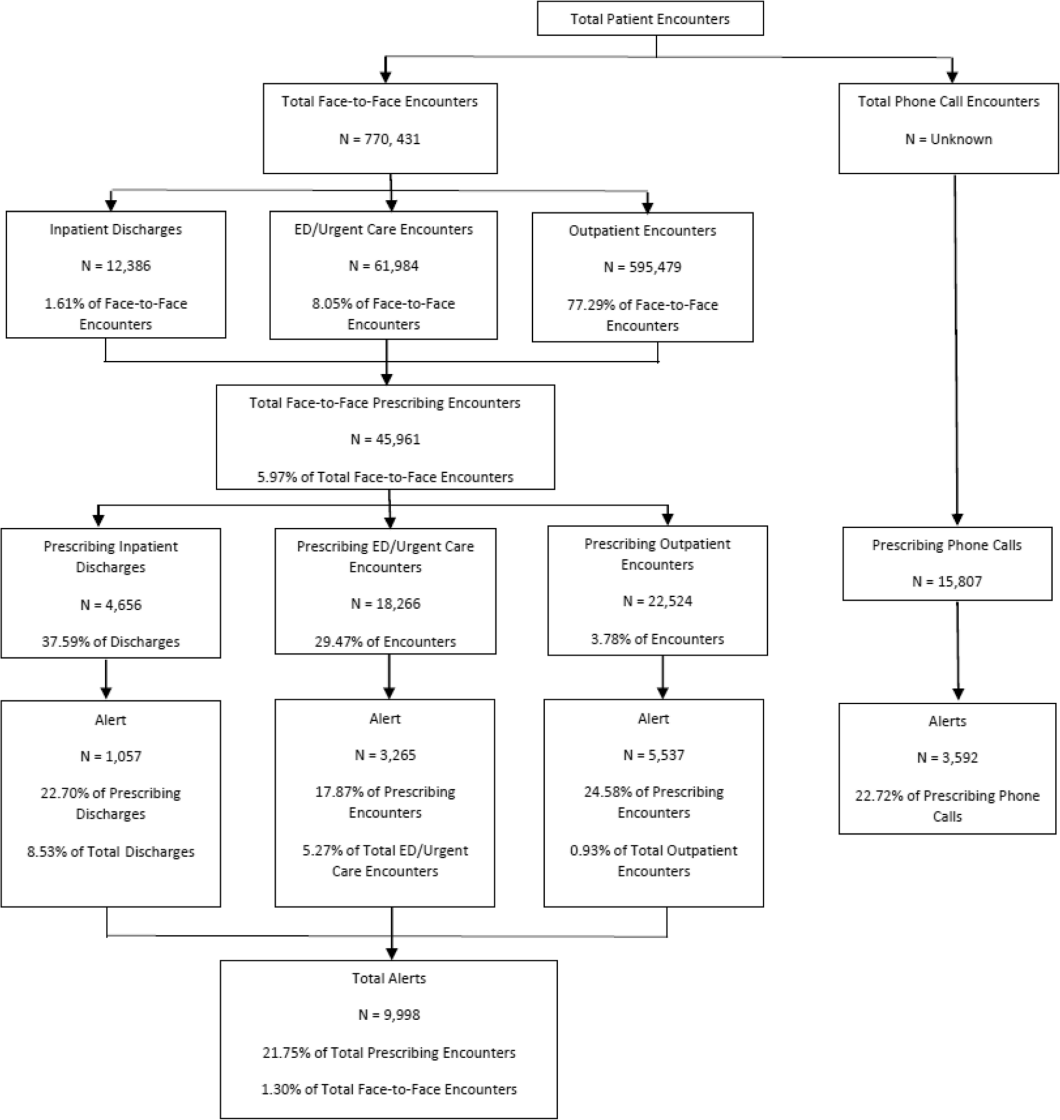
Rate of Narcotic/Benzodiazepine Prescription Encounters and Rate of PRIMUM Alert *Note: Due to lack of documentation of phone call encounters and, therefore, inability to generate an accurate total denominator for patient phone calls requesting prescriptions, these data were separated in this figure

The rates of alerts system-wide and by facility type (inpatient, ED/Urgent Care, and outpatient) are presented in Fig. 2. A total of 9998 alerts would have been generated during the month of data collection if the alert were not in “silent” phase. Overall, once live, the alert would have fired in 5.97 % of encounters where a prescription for an opioid or benzodiazepine was written and in only 1.30 % of all face-to-face patient encounters. Alerts would have fired most frequently at inpatient discharges (8.53 % of total discharges) and least frequently in the outpatient setting (0.93 % of patient encounters). These rates indicate the alert will have a low burden on prescribers, yet suggest that they will capture a significant number of at-risk patients, yielding substantial public health impact.

### Prescription utilization mapping

The characteristics of prescribing encounters from *Silent Surveillance 2* are presented in Table 3. The volume of prescriptions varies by location, with the majority of prescribing encounters occurring in outpatient facilities (62 %). However, the distribution of prescribing encounters among facility types does not match the distribution of overall patient encounters. As expected, inpatient discharges and ED/Urgent Care encounters have disproportionately higher rates of opioid and benzodiazepine prescriptions compared to the number of patient encounters. While inpatient discharges make up less than 2 % of encounters, they represent 7.5 % of prescribing encounters. Similarly, ED/Urgent Care encounters represent less than 10 % of total encounters, yet 29.6 % of prescribing encounters. While the majority of opioid and benzodiazepine prescriptions are written to patients 18–64 (73.80 %), 23.7 % are written to older adults (≥65 years of age), and 2.5 % to children (≤17 years of age). Opiates are prescribed more frequently (73.2 %) than benzodiazepines, and 3.8 % of encounters resulted in co-prescribing opiates and benzodiazepines.

The most common trigger is a current prescription with >50 % remaining, present in 13.54 % of prescribing encounters, while previous presentation for overdose is the least prevalent (0.81 %). The alert would have fired in 22.00 % of prescribing encounters. While the majority of patients did not meet any criteria (78.00 %), 4.97 % of prescribing encounters were with patients meeting multiple risk criteria.

## Discussion

Prescription opioid and benzodiazepine use has reached epidemic proportions in the United States. Through literature review and consensus building with a multidisciplinary team, we identified five objective criteria (See Final Triggers) that indicate high risk for misuse, abuse, and diversion of prescription opioids and benzodiazepines and built an alert system in the electronic medical record of a large healthcare system. We found that a significant number of patients meeting these criteria are being prescribed opioids or benzodiazepines, indicating risk for misuse, abuse, and diversion. However, the alert will fire in a small percentage of patient encounters, and thus should not be intrusive to prescribers’ workflow.

We are not aware of any previous studies that have used this process to prospectively develop and test interventions in the EMR. However, there are examples in the literature of utilizing an intensive process of modeling clinical data “warehouses” for refining alerts related to medication orders [46, 47]. These previous authors utilized clinical data to model outcomes resulting from various thresholds set for medication alerts. The method we present in this paper is a novel, and another, approach to the development, testing, and implementation of clinical decision support in the EMR. The combination of expert panel and literature review coupled with “silent” phases to properly test and tune the triggers using “live” data is a novel approach that can be utilized in the development of future EMR-based interventions. These two “silent” alert phases were extremely useful, both for obtaining baseline data and to test the alert prior to launching this intervention. Similar testing of real clinical data in a non-clinical test environment has been utilized in other electronic medical record settings [48]. The data collected allowed us to choose triggers that did not cause the alert to fire too often, but successfully captured the at-risk population. This was important to minimize the potential for alert fatigue. These data were also helpful in obtaining support for deployment of this intervention within the healthcare system by being able to illustrate the problem, as well as provide evidence that the alert would be minimally obtrusive.

This intervention is currently being implemented in a single, although very large, healthcare system, which limits the available information about prescriptions or past medical history to encounters inside this system. Therefore, future interventions will aim to link to the state-wide prescription drug monitoring program to obtain a complete prescription history for each patient, as well as expand to other regional healthcare systems. In addition, we acknowledge that alerts do not exist in a vacuum and, while PRIMUM may not be that intrusive to prescribers, we are unable to determine the number of alerts a given prescriber might see for a given patient outside of the alert for PRIMUM, or the number of alerts seen during a day or clinical shift. Collaboration and coordination within a large healthcare system to address clinical decision support is needed. Future research will analyze the prescriber behavior related to the alert as well as the appropriateness of these triggers for subpopulations, such as cancer, chronic pain, or palliative care patients. The data on specific populations will also be used to develop targeted interventions to assist prescribers and patients once risk is identified.

## Conclusions

Future initiatives in healthcare systems should utilize the capacity of their EMR to collect data prospectively to inform interventions. Given the complexity of a large healthcare system, it is important that EMR alerts are tested and tuned sufficiently to ensure a smooth rollout and that buy-in and support from all stakeholders is obtained early in the process. The utilization of the silent phases was a useful step that is translatable to other EMR systems.

## Abbreviations

EMR: electronic medical record
PDMP: prescription drug monitoring program
PRIMUM: Prescription Reporting with Immediate Medication Utilization Mapping
