# Erratum to: Prescription reporting with immediate medication utilization mapping (PRIMUM): development of an alert to improve narcotic prescribing

**DOI:** 10.1186/s12911-016-0364-6

**Published:** 2016-09-26

**Authors:** Rachel B. Seymour, Daniel Leas, Meghan K. Wally, Joseph R. Hsu, the PRIMUM Group, Michael Beuhler, Rebecca Boland, Michael J. Bosse, Stephen Colucciello, Emily Gerkin, Michael Gibbs, Christopher Griggs, Anthony Jacobson, Steven Jarrett, Jan Losby, Monica Mowry, Michael Runyon, Animita Saha, Sharon Schiro, Bradley Watling, Claire Wedgworth, Stephen Wyatt

**Affiliations:** 1 Department of Orthopaedic Surgery, Carolinas Health Care System, 1000 Blythe Boulevard, Charlotte, 28203 NC USA; 2 Carolinas Trauma Network Research Center of Excellence, Carolinas Health Care System, 1320 Scott Avenue, Charlotte, NC 28204 USA

## Erratum

Following the publication of the original article [1] it was brought to our attention that the collaborators from the PRIMUM Group were not correctly marked up as collaborating authors. This has now been corrected and all authors should be searchable in PubMed. The group consists of the following authors: Michael Beuhler; Rebecca Boland; Michael J. Bosse; Stephen Colucciello; Emily Gerkin; Michael Gibbs; Christopher Griggs; Anthony Jacobson; Steven Jarrett; Jan Losby; Monica Mowry; Michael Runyon; Animita Saha; Sharon Schiro; Bradley Watling; Claire Wedgworth; Stephen Wyatt.
